# galign: A Tool for Rapid Genome Polymorphism Discovery

**DOI:** 10.1371/journal.pone.0007188

**Published:** 2009-09-25

**Authors:** Shai Shaham

**Affiliations:** Laboratory of Developmental Genetics, The Rockefeller University, New York, New York, United States of America; The University of Chicago, United States of America

## Abstract

**Background:**

Highly parallel sequencing technologies have become important tools in the analysis of sequence polymorphisms on a genomic scale. However, the development of customized software to analyze data produced by these methods has lagged behind.

**Methods/Principal Findings:**

Here I describe a tool, ‘galign’, designed to identify polymorphisms between sequence reads obtained using Illumina/Solexa technology and a reference genome. The ‘galign’ alignment tool does not use Smith-Waterman matrices for sequence comparisons. Instead, a simple algorithm comparing parsed sequence reads to parsed reference genome sequences is used. ‘galign’ output is geared towards immediate user application, displaying polymorphism locations, nucleotide changes, and relevant predicted amino-acid changes for ease of information processing. To do so, ‘galign’ requires several accessory files easily derived from an annotated reference genome. Direct sequencing as well as *in silico* studies demonstrate that ‘galign’ provides lesion predictions comparable in accuracy to available prediction programs, accompanied by greater processing speed and more user-friendly output. We demonstrate the use of ‘galign’ to identify mutations leading to phenotypic consequences in *C. elegans*.

**Conclusion/Significance:**

Our studies suggest that ‘galign’ is a useful tool for polymorphism discovery, and is of immediate utility for sequence mining in *C. elegans*.

## Introduction

The advent of high throughput sequencing has allowed researchers to generate copious amounts of sequence data, raising the possibility that whole genome sequencing (WGS) may become a viable method for identifying sequence polymorphisms leading to phenotypic consequences. Indeed, a recent publication [1] demonstrated the use of WGS, in conjunction with genetic mapping, for identifying the sequence lesion responsible for defects observed in *lsy-12* mutants of *Caenorhabditis elegans*. A similar strategy has allowed causal lesions in a different *C. elegans* mutant to be identified as well (G. Oikonomou and S. Shaham, unpublished observations). High throughput sequencing approaches generally rely on sequencing of short DNA fragments (30–300 base pairs) derived from a genome under investigation. For extensive coverage of the genome, therefore, a large number of sequence fragments must be read. For example, for a genome of size 100 Mbp and using sequence fragments of length 32 nucleotides (nts), 21,586,731 independent sequence fragments must be read theoretically to achieve a 99.9% probability that a given genomic position is sampled at least once. This number is generally a lower limit, as various biases and sequence errors often reduce the quality of sequence reads. For single nucleotide polymorphism (SNP) detection, 3 sequence reads of a given position are often used as a minimal quality standard. In such cases, the number of sequence reads required to ensure a 99.9% probability of detection doubles.

Fortunately, such extensive parallel sequencing is easily achievable using current technologies. One major sequencing technology, Illumina/Solexa, can now generate over 100 million sequence reads of up to 75-nt each in a single run of eight sequencing lanes, allowing the medium-sized genomes of *C. elegans* and *Drosophila* to be rapidly interrogated for SNPs. In this paper we primarily focus on the analysis of sequences derived from the *C. elegans* genome. We therefore define a “standard sequencing experiment” as 40 million 32-nt reads of this genome (100 Mbps)- this represent roughly 12× coverage, and is sufficient to guarantee, at least in theory, that essentially all positions are sampled.

Computational tools to mine sequencing data for SNP discovery have lagged behind the sequencing technologies themselves. However, a number of recent efforts have led to the generation of software for this purpose, including the free programs ‘Maq’ [2](http://maq.sourceforge.net/), ‘SSAHA’ [3](http://www.sanger.ac.uk/Software/analysis/SSAHA/), ‘GMAP’ (http://www.gene.com/share/gmap/), ‘Mosaik’ (http://bioinformatics.bc.edu/marthlab/Mosaik), ‘Bowtie’ [4](http://bowtie-bio.sourceforge.net/index.shtml), and ‘SOAP’ [5](http://soap.genomics.org.cn/); and the proprietary software ‘SXOligoSearch’ (http://synasite.mgrc.com.my:8080/sxog/NewSXOligoSearch.php) and ‘Eland’ (http://www.illumina.com). ‘SSAHA’, ‘GMAP’, and ‘Mosaik’ are all-purpose sequence alignment programs, not necessarily optimized for SNP discovery. ‘SXOligoSearch’ is similarly designed to align short sequences to a genome. ‘SOAP’, ‘Bowtie’, ‘Maq’, and ‘Eland’ are geared towards polymorphism discovery. A direct comparison of all these programs has not been published, however, recent advances based on the Burrows-Wheeler Transform and FM-indexing used by ‘Bowtie’ have reported achieving speeds significantly faster than ‘Maq’ and ‘SOAP’ [4], and allowing an order of magnitude reduction in memory usage, suggesting that much optimization is left to pursue. Anecdotal reports suggest that ‘Eland’ can achieve similar speeds, but with a larger memory footprint. For all these alignment programs considerable user-designed secondary processing is necessary to arrive at a list of reliable SNPs and their properties.

Here I describe software, named ‘galign’, intended to identify SNPs and other alterations with respect to a reference genome using sequence reads obtained by Illumina/Solexa sequencing. The software is aimed at identifying candidate sequence lesions leading to an observable mutant defect in the organism under investigation. ‘galign’ source code is written in C++, and the executables available for download are currently for the Mac OSX Intel platform. Unlike other alignment tools, the basic ‘galign’ alignment tool does not use the Smith-Waterman algorithm [6]. Instead, parsing of genomic and read sequences followed by positional proximity of matched sequences is used to map reads onto the genome in a manner superficially reminiscent of SSAHA alignments [3]. The software is particularly useful for small and medium-sized genomes and has been extensively tested on the heavily annotated genome of *Caenorhabditis elegans* (www.wormbase.org). Application to larger genomes is possible, but requires adequate memory capacity and allocation (see Results).

‘galign’ software is easy to use and, importantly, displays SNP and alteration calls in an accessible format eliminating the need for user-dependent secondary processing. In addition, alignment and post-alignment processing are rapid. Speed and output comparisons of ‘galign’ to ‘Maq’ suggest that ‘galign’ is a competitive alternative to existing SNP detection tools.

## Results

‘galign’ consists of a suite of applications independently executed by the user to obtain polymorphism data. Following conversion of sequence data to the ‘galign’ format, raw data generated by the alignment tool, Alignment_tool, is processed using a nucleotide substitution detection tool, SNP_search, and a tool for detecting other alterations, Deletion_search. Below we describe each program.

### The ‘galign’ Alignment Algorithm

#### Parsing the genome

‘galign’ uses two input files for polymorphism detection: a reference genome sequence file in which all chromosome sequences are concatenated in order, and a sequence-reads file, containing consensus Illumina/Solexa sequence reads on separate lines. The genome sequence file is assembled into the required format using the provided concatenation application Genome_assemble, which uploads individual chromosome sequences in Fasta format and converts them into a single ‘galign’-compatible file. The sequence-reads file can be extracted from Fasta, Fastq, or gseq Illumina output formats using the Format_convert tool. Alignment begins with the genome sequence being read in groups of 13 consecutive nucleotides starting at one end of the genome sequence, and advancing one nucleotide at a time. Based on our initial testing, a length of 13 nts offered the best compromise between speed and memory allocation, both of which increase with length (see below). Each genome position is assigned to a number array (the genome position array) whose element number is the genomic position, and whose value is a unique numerical descriptor of the corresponding 13-nt sequence. The descriptor is a base 4 representation of the 13-mer generated by the formula 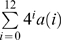, where *a(i)* takes on the values *0*, *1*, *2*, or *3* if the nucleotide at position *i* reads *G*, *A*, *T*, or *C* respectively [3]. Next, a set of 4^13^ number arrays (pointer arrays) are generated. Each pointer array corresponds to a possible base 4 value of a 13-nt sequence, and the number of elements in each array corresponds to the number of occurrences of a given 13-nt sequence in the genome. The value of each array element is the genomic position of each 13-nt sequence.

#### Aligning a wild-type sequence read

**Figure 1:**
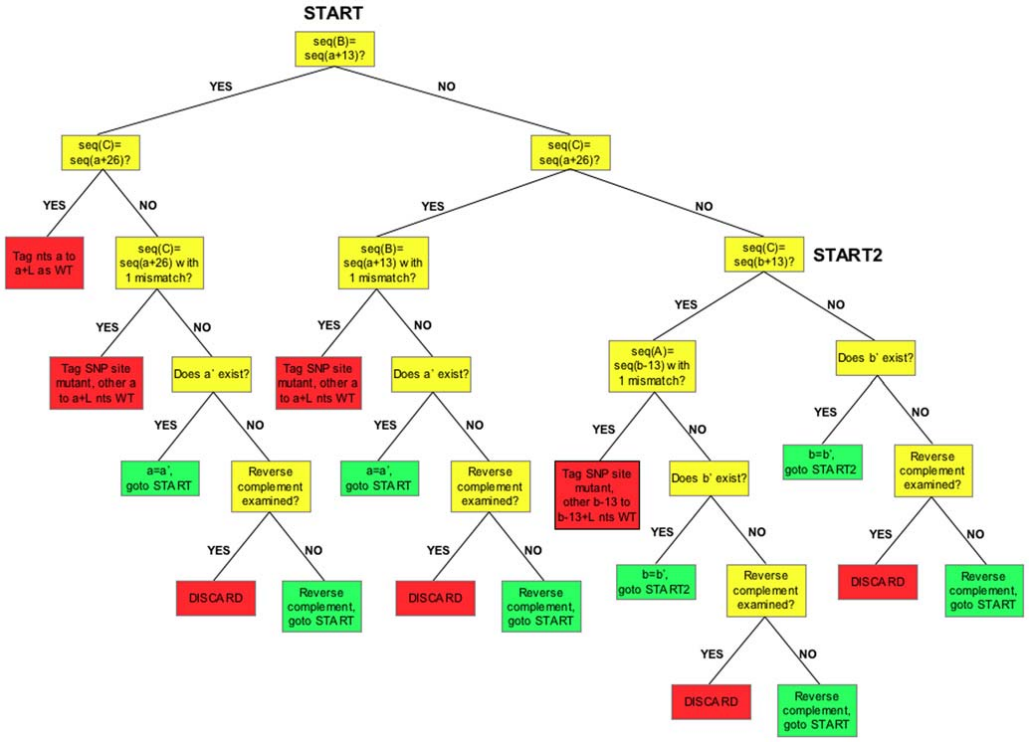
‘galign’ alignment algorithm. A sequence read is divided into three fragments, *A*, *B*, and *C* (see Results). Algorithm starts at START. seq(A), the sequence of fragment *A*. seq(a), a genomic sequence matching seq(A) and located at position ‘a’ in the genome. a', an alternate genomic location containing seq(A). L, length of sequence read. Yellow boxes, decision nodes. Green boxes, algorithm repeat nodes. Red boxes, algorithm end points.

Once the genome sequence has been parsed, ‘galign’ loads the first sequence read from the sequence-reads file into memory and divides it into 3 segments, *A*, *B*, and *C*, of respective lengths 13, 13, and *x*, with 0*≤x≤*13. ‘galign’ then checks whether sequence *A* exists in the genome, by referring to the pointer array corresponding to sequence *A*. If the sequence is found, the first genomic position of *A* is noted, and the program asks whether sequences *B* and *C* exist at positions 13 and 26 nts downstream of the genomic position of *A*, respectively, by referring to the pointer arrays (see Figure 1 for a detailed flow chart of the algorithm). If fragment *C* is shorter than 13 nts, as is generally the case, the appropriate length genomic sequence is extracted from the genome position array value. If matches are found, a value of 1 is added to a new number array (the wild-type array) whose element numbers correspond to positions in the genome, and whose values represent the number of times that position has been read as wild type.

For sequence reads longer than 39 nts and up to 78 nts, a beta version of ‘galign’ is available in which Format_convert will generate two overlapping reads from each input sequence in such a way as to minimize overlap. Each read is then searched by the alignment tool independently. A correction is introduced in the alignment tool to prevent counting overlapping residues twice. For sequence read lengths of 51–78 nts, the alignment tool runs at essentially maximal efficiency, as overlap between reads is either 0 or 1 nt. For read lengths of 39–50 nts, efficiency is compromised by an increase in overlap, as sequence length decreases. This loss of efficiency, however, is counter-balanced by each read being only 26 nts long.

#### Aligning a sequence read with one nucleotide substitution

Single substitutions could occur in either fragment *A*, *B*, or *C*. If a match to the genome with fragment *A* is not detected, ‘galign’ attempts to match sequences *B* and *C* next to each other in the genome. If this is successful, the program revisits sequence *A* and examines whether genomic sequences 13 nts upstream of sequence *B* can be matched to sequence *A* with one substitution. Single substitutions are detected by subtracting the base 4 representation of the genomic sequence from that of the sequence read and only allowing a change of a multiple of a single power of 4 (see above).

If sequence *A* is matched in the genome but *B* is not, ‘galign’ checks to see if fragment *C* can be exactly aligned. If *C* matches, sequence *B* is tested for having a single substitution as above. A similar procedure is followed for putative substitutions in fragment *C* (Figure 1).

Finally, if single substitution matches are not found, ‘galign’ queries deeper elements of the pointer arrays and repeats the process for other genomic sites containing the *A* or *B* fragments. If no matches are detected at these positions, ‘galign’ generates the reverse complement of the input fragment, and repeats the above algorithm. If an alignment of a single-mismatched read is made, a value of 1 is added to each element of the wild-type array in which a sequence match was detected. A value of 1 is also added to an element of a new number array (the mutant array) corresponding to the position of the mismatched residue in the genome. In addition, the nucleotide sequence of a mutant 13-nt fragment, starting at the mutation site, and of the corresponding wild-type sequence, deduced from the genomic sequence, are added to two new character arrays (the mutant and wild-type sequence arrays, respectively) whose elements correspond to the position of the mutation site in the genome.

#### Aligning a sequence read with more than one nucleotide substitution, a deletion, or an insertion

‘galign’ is designed to provide a low false positive rate of polymorphism prediction. To do so, the alignment algorithm is conservatively designed to process sequence reads with at most one mismatch (although this can be easily changed within the alignment tool, and may become an option in future releases). Regions in which true multiple local substitutions occur, or that contain insertions or deletions, and which cannot be covered by single-mismatch sequence reads can, however, be detected by their absence using the Deletion_search tool described below. It is noted that our studies of data sets from ethyl-methanesulfonate (EMS) mutagenized *C. elegans* strains suggest that single mismatch detection is sufficient for polymorphism detection. Indeed, although additional calls may be generated by allowing more mismatches in the alignment, the false positive rate also increases (see below), offsetting gains in predictive power.

#### Output

**Figure 2:**
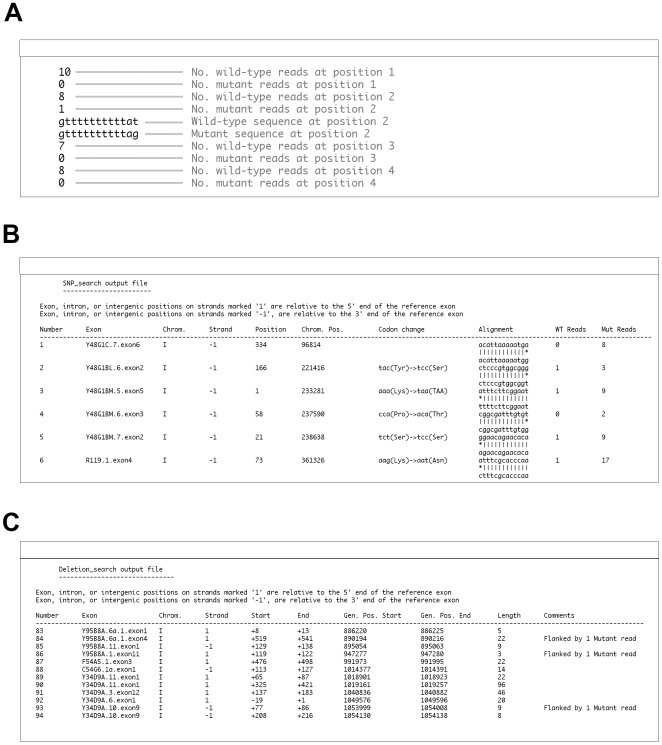
‘galign’ output files. (A) A portion of a ‘galign’ alignment tool output file indicating the numbers of wild-type and mutant reads at a given position, as well as the corresponding wild-type and mutant sequences displayed in the event that a mutation was detected. (B) A portion of a SNP_search output file is depicted for a search involving exonic sequence substitutions. Position, position with respect to exon start site. Chrom. Pos., position with respect to the indicated chromosome. WT reads, number of wild-type reads at the given position. Mut reads, number of mutant reads at the given position. (C) A portion of a Deletion_search outputfile is depicted for a search involving deletions spanning exons. Start, End, the start and end coordinates of the deletion with respect to the first nucleotide of the indicated exon. Gen. Pos. Start, Gen. Pos. End, start and end coordinates of deletion with respect to the indicated chromosome. The Comments column is used to highlight features indicative of true deletions and insertions.

After scanning through all sequence reads, the alignment tool generates a file containing the collected information. The file begins at position 1 of the first chromosome, displaying the value of the wild-type number array for that position, followed by the value of the mutant number array corresponding to that position. If the mutant number array value is not 0, the wild-type and the last mutant sequence stored in the respective sequence arrays are displayed (Figure 2A).

#### Memory

For a genome of 100 Mbps, ‘galign’ requires 4*100,000,000 = 400,000,000 bytes for the genome array, and about 400,000,000 bytes for the pointer arrays, wild-type, and mutant number arrays. Memory allocation for the sequence strings is usually relatively small, unless the number of mutations is of the order of the number of nucleotides in the genome. Thus, roughly 1.6 Gb of memory needs to be allocated. It may be possible to somewhat reduce memory expenditure by using hash tables instead of arrays. Thus, ‘galign’ achieves speed (see below) at the expense of memory.

### Detecting Single Nucleotide Substitutions

Nucleotide substitution data can be extracted from the alignment output file using the SNP_search tool. The output of SNP_search is a text file containing an ordered list of predicted nucleotide substitutions characterized by their positions with respect to a reference exon and with respect to the chromosome sequence, the predicted sequence alteration, and in the case of lesions in coding regions, the predicted amino-acid substitution (Figure 2B). The numbers of wild-type and mutant reads giving rise to the prediction are also displayed.

**Figure 3:**
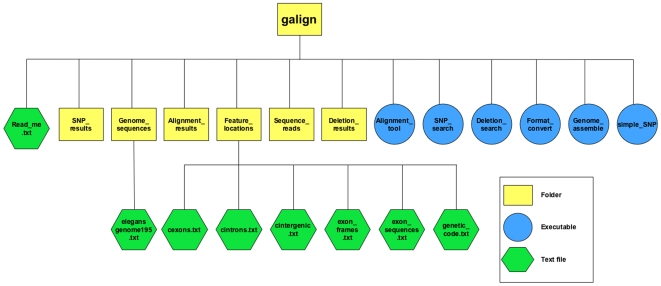
File structure of the ‘galign’ folder. Yellow boxes, folders. Blue circles, executable files. Green hexagons, accessory text files. SNP_results contains the output of SNP_search. Alignment_results contains the output of Alignment_tool. Sequence_reads contains the output of Format_convert. Deletion_results contains the output of Deletion_search. The output of Genome_assemble is located in the Genome_sequences folder. A pre-assembled *C. elegans* genome (version 195) is distributed with the current software package. Feature_locations contains information about exons, introns and intergenic regions (see text) as well as the genetic code table for amino-acid predictions.

To perform its tasks, SNP_search requires five accessory files: a file containing a list of exon names, exon start and end positions, chromosomal locations and strand information sorted by chromosome and position; two similar files for introns and intergenic regions, referenced by the upstream exon in the case of the intron file, and by the downstream exon for the intergenic region file; a file containing a list of exon names, the positions of the start and end of coding regions in each exon, and the reading frame (1, 2, or 3) at the 5′ most nucleotide of the exon (unless it is the first exon of a gene, in which case the 5′ reading frame is assigned the value 1); and a file containing a list of exon names and their sequences. For the *C. elegans* genome, these data are readily available at http://www.wormbase.org and are prepackaged together with the concatenated genome sequence in the current release of ‘galign’ (see Materials and Methods; Figure 3).

Before executing SNP_search, the user is instructed to input three parameters restricting SNP detection: the minimum ratio of the number of mutant to wild-type reads required for calling a SNP, the minimum number of mutant reads to call a SNP, and whether SNPs should be scanned in exons, introns, or intergenic regions. The default settings for the first two parameters are 3 and 2, respectively. Thus, a position with 0 wild-type reads will be called a SNP if at least 2 mutant reads are detected at that position (a total of 2 reads for that position); however, positions with one or more wild-type reads would require a minimum of 4 reads in total.

SNP_search then scans the alignment output file, noting all positions satisfying the criteria set by the user. Flagged positions are queried as to whether they fall within exons, introns, or intergenic regions, as indicated by the user, by comparing SNP position in the genome with the list of exon/intron/intergenic region start and end positions. SNP_search broadens the definition of an exon to include 10 nucleotides upstream and downstream of an exon, to facilitate identification of mutations that disrupt splice sites. Since the program only stores in memory genomic positions matching the filtering criteria, memory usage by the program is usually quite small.

A companion application, simple_SNP, intended for genomes that have not been annotated is also available. This application processes the Alignment_result file to filter out sequence not meeting specified user criteria. simple_SNP output does not require genome annotation files and does not indicate whether an interrogated position falls in an intron, exon, or intergenic region (Figure 3).

For exonic domains, SNP_search next assesses whether the SNP falls inside a coding region. If so, the amino-acid affected by the SNP is recovered by subtracting the SNP position from the exon start position, calculating the resulting value modulo 3, and assessing the frame with respect to the known frame at the exon start site. The appropriate codon is then retrieved from the exon sequence files either directly or by stitching sequences from adjacent exons in the event that a SNP affects either the first two or the last two nucleotides of an exon. Both wild-type and mutant amino acids are then printed to the output file. For introns and intergenic regions, SNP_search outputs a file similar to that presented in Figure 2B, except that the column displaying codon changes is absent.

### Identification of deletions, insertions, and closely-spaced substitutions

The detection of deletions, insertions, and multiple local substitutions has proven a major challenge for all SNP discovery tools described to date. Below, we consider several possible lesions and describe their detection by ‘galign’.

#### Deletions greater than one nucleotide

The Deletion_search tool of ‘galign’ identifies all positions in the genome that have no sequence reads, wild-type or mutant, as defined by the alignment tool output file. In addition to the output file of the alignment program, Deletion_search also requires 3 accessory files containing the names, start and end positions, chromosome, and strand locations of exons, introns, and intergenic regions sorted by chromosome and position. The program searches through the alignment output file for an occurrence of 0 wild-type and 0 mutant reads. Once such a position is encountered, a running counter measures the length of the unread region, until a position with non-zero reads is reached. The start and end point of each unread region is then compared to exon/intron/intergenic region start and end sites to determine whether the region spans the user-indicated feature set. The output file produces an ordered list, based on chromosome position, of all genomic locations that lack hits. The start and end positions of each unread region are displayed with respect to the relevant exon and chromosome, as are the lengths of each unread region (Figure 2C).

**Figure 4:**
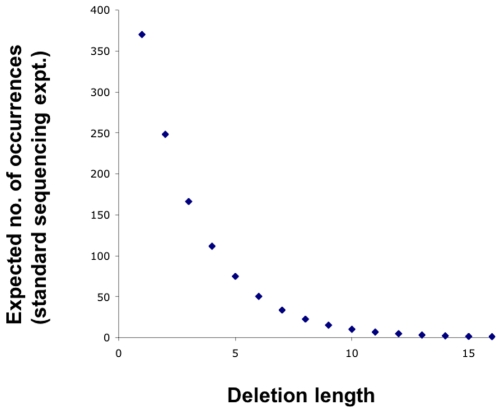
Expected stochastic occurrences of unread genomic sequence stretches. The number of expected unread sequences of a minimum length is plotted for a genome of size 100 Mbps and 40 million 32-nt sequence reads. The probability, *P*, of obtaining an unread sequence of at least length *L* is equal to the probability of not obtaining any 32-bp sequence fragments that cover a stretch of length *L*. This is given by 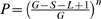, where *G* is the genome size, *S* is the sequence read length (32), and *n* is the number of sequence reads examined. The expected number of deletions is then given approximately by 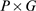.

Long stretches of unread genomic regions arising from sampling error of the genome are predicted to be exceedingly rare for a standard sequencing experiment. As shown in Figure 4, stochastic unread sequence stretches of 15 nts or more are theoretically predicted to occur less than once per genome. Thus, large stretches of unread sequences are likely to arise from three possible situations: First, since the ‘galign’ alignment tool assigns all repetitive sequences to the first occurrence of the sequence in the genome, and repeats occurring more than four times in the genome are ignored, large unread stretches could correspond to repetitive domains of the genome. We have verified this in the case of telomeric sequences (data not shown). Second, DNA regions whose amplification in sequencing reactions is not favored may not be represented in the sequence reads. Third, true alterations in the sequenced genome, with respect to the reference genome, may account for large unread domains.

**Figure 5:**
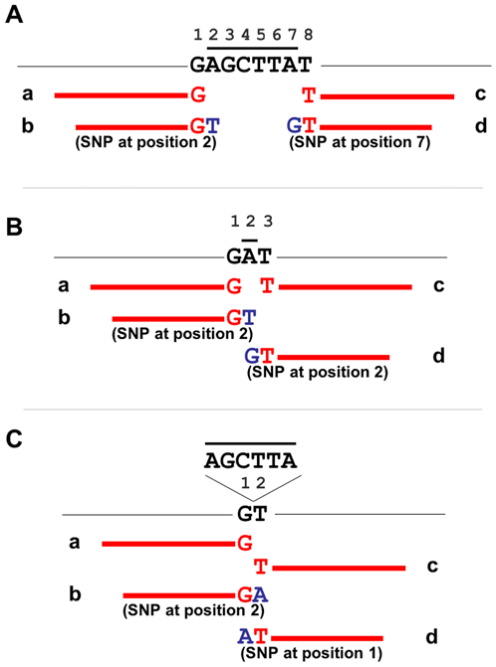
Deletions and insertions can be detected using SNP_search. (A) The genome sequence is indicated by a thin black line, with eight genomic nucleotides, GAGCTTAT, explicitly shown. Nucleotides are arbitrarily numbered above the sequence from 1 to 8. Thicker black bar, nucleotides deleted in the genome from which the sequence reads were generated. Red lines, sequence reads, where the terminal nucleotides of the read are indicated either in red or blue. Blue nucleotides indicate that the nucleotide is not contiguous with the adjacent red nucleotide in the reference genome, but is adjacent to the red nucleotide in the genome being sequenced. Four sequence reads derived from the genome being sequenced and flanking or traversing the deletion site will be recognized by ‘galign’. Sequences ‘a’ and ‘c’ will flank the deleted region, whereas sequences ‘b’ and ‘d’ will span the deletion. For sequence ‘b’, nucleotides 1 and 8 of the reference genome are adjacent in the sequenced genome because of the deletion of nucleotides 2–7. ‘galign’ will read this as a mismatch at position 2 of the reference genome. Likewise, for sequence ‘d’, ‘galign’ will read a mismatch at position 7 of the reference genome. Occasionally, deletion-spanning reads will happen to have the same sequence as the reference genome lacking the deletion. This will not change the analysis described in the text because a deletion-spanning read can (nearly) always be found that will be called as a mismatch. (B), same as A, except that a single nucleotide deletion is depicted. (C), same as A, except that an insertion is depicted.

Distinguishing among these options is not always possible. However, if coverage of the genome is extensive, runs of unread regions corresponding to true deletions should be flanked by single or, more rarely, more than one position containing at least one mutant read. This occurs because 32-nt sequence fragments that span the deletion site will be read as a mismatch by the alignment tool (see Figure 5A). For a specific deletion, the probability of finding such a mismatched sequence read on at least one side of the deletion can be calculated to a first approximation as 
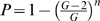
, where *G* is the genome size, *n* is the number of sequence reads, and 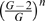 is the probability of not finding any mismatched sequence reads. For a standard sequencing experiment, *P* is about 55%. Deletion_search flags sequences containing such flanking regions in the output file under the “Comments” heading.

An upcoming release of ‘galign’ will also incorporate positive data from sequence reads spanning deleted or inserted sequences. Comparison of these data with those provided by Deletion_search should make for more accurate deletion/insertion calls.

The Deletion_search tool will tend to overestimate deletion sizes. Each genomic position can, in principle, be covered by 32 different 32-nt sequence reads. Thus, the probability of reading a given position in a standard sequencing experiment is theoretically 99.9997%. However, using the ‘galign’ alignment tool, a genomic position directly abutting a deletion sites can only be covered by two sequence reads (and more rarely by a larger number). The detection probability of such a position is also 
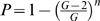
, reducing coverage to only about 55% per site for a standard sequencing experiment. This expansion of deletions has been verified experimentally (see below).

Overall, therefore, the vast majority of deletions greater than one nucleotide are predicted to be found by ‘galign’ using the Deletion_search tool, and some of these will possess signatures distinguishing them from stochastic read failures or repetitive sequences.

#### Single nucleotide deletions

Unlike large deletions, there is a significant probability that single nucleotide deletions will not be detected as unread genomic regions by the Deletion_search algorithm. Specifically, such deleted positions could be covered by two (or, rarely, more) possible sequence reads, giving a coverage probability of at least 55% (Figure 5B; see above). Deletions that are covered by reads will generate a mutant read at the deleted nucleotide position in 15/16 of such sites (Figure 5B), or will have a probability of 55% of generating a SNP in the vicinity of the deleted nucleotide in the remaining 1/16 of sequences. Overall, therefore, 98% of single nucleotide deletions will generate either no reads or at least one mutant read in the vicinity of the deletion in a standard sequencing experiment. Whether all deletions are actually detected by the user is, of course, heavily dependent on the parameters used for nucleotide substitution calls.

#### Insertions

Detection of insertions by ‘galign’ follows similar considerations to deletion detection, however, unlike deletions, whose size can be estimated, insertion size is either difficult or impossible to extract from the ‘galign’ alignment output (although with the incorporation of lesion-spanning reads in the upcoming ‘galign’ release, sizes of small insertions will be measurable). As with single nucleotide deletions, insertion sites will nearly always generate either no reads or a mutant read in the vicinity of the insertion (Figure 5C). For a standard sequencing experiment, about 28% of insertions are predicted to have a unique signature of two adjacent positions containing mutant reads (Figure 5C). Although such dyads can arise from independent events, they may be an indication of insertion sites, and are displayed in the “Comments” column of the Deletion_search algorithm output.

#### Multiple local substitutions

Although the presence of two or more nucleotide substitutions within a 32 nt region could be revealed by SNP_search, these regions may also be detected by Deletion_search, since most sequence reads spanning these areas will contain more than one mismatch, and will not be read by the alignment tool, giving rise to unread positions. Distinguishing such instances from true deletions may not be possible in ‘galign’. It is worth noting, however, that lesions predicted by polymorphism-detection tools only serve as guides for additional analysis using direct sequencing. Thus, in some respects, the exact nature of a lesion may be less important than the observation that a lesion exists.

### Experimental Validation of ‘galign’ Predictions

To test the ability of ‘galign’ to predict polymorphisms, we examined two Illumina/Solexa sequence read data sets from EMS-mutagenized *C. elegans* strains. The first data set was generated from a strain carrying two mutations with known phenotypic consequences: one in the *daf-6* gene (*e1377*) [7], and another in an unknown suppressor of the *daf-6* mutation (G. Oikonomou., E. Perens, and S. Shaham, unpublished observations), and consisted of 42 million sequence reads. The second data set was kindly provided by Dr. Oliver Hobert and was generated from a mutagenized *C. elegans* strain containing the *lsy-12*(*ot177*) mutation [1]. This data set consisted of 54 million sequence reads. Both strains were compared to the *C. elegans* reference genome (release 195).

We sequenced 12 sequence polymorphisms predicted by ‘galign’ from the *daf-6* data set. 11/12 corresponded to sequences that were indeed different from the reference wild-type *C. elegans* (N2) strain (Table 1). Of these, 10/11 polymorphisms were nucleotide substitutions, and 1/11 was a deletion. For the deletion polymorphism, the true length of the deletion was 99 nts, whereas the ‘galign’ predicted length was 122 nts (data not shown), consistent with our prediction that the Deletion_search tool will tend to overestimate deletion size. 1/12 lesions examined did not show differences from the wild type, and may correspond to a systematic sequencing error introduced during the sequencing process or an alignment failure. Finally, one of the 11 polymorphisms we confirmed was the polymorphism leading to the *daf-6*(*e1377*) mutant phenotype, showing that ‘galign’ was able to detect a lesion causally related to an underlying defect.

**Table 1:** Assessing ‘galign’ predicted polymorphisms by direct sequencing.

Experiment	Position	Chromosome	Associated gene	Wild-type nucleotide	Nucleotide predicted by ‘galign’	Confirmed by direct sequencing?
*daf-6*(*e1377*)	1130637	X	*snx-1*	N/A	Deletion	**Yes**
	2846639	X	*kin-29*	A	G	**Yes**
	3056766	X	*tag-312*	A	G	**Yes**
	6376965	X	*lmp-1*	T	G	**No**
	7566550	X	*tag-197*	C	T	**Yes**
	7588882	X	*adt-2*	C	T	**Yes**
	10783754	X	*clc-4*	A	G	**Yes**
	11377242	X	*tni-1*	A	G	**Yes**
	11624628	X	*twk-44*	C	T	**Yes**
	12337564	X	*srd-48*	A	G	**Yes**
	12798779	X	*gei-3*	T	A	**Yes**
	14889350	X	*daf-6*	G	A	**Yes**
*lsy-12*(*ot177*)	8684610	V	*cln-3.3*	G	G	Yes
	8684630	V	*cln-3.3*	G	G	Yes
	8571627	V	*F25G6.2*	G	T	**Yes**
	9217870	V	*grd-6*	A	G	**Yes**
	8684649	V	*cln-3.3*	T	T	Yes
	9218398	V	*grd-6*	C	T	**Yes**
	9245971	V	*aat-2*	C	T	**Yes**
	9986752	V	*C27H6.8*	G	A	**Yes**
	8684663	V	*cln-3.3*	T	T	Yes
	8684570	V	*cln-3.3*	G	G	Yes
	9846725	V	*lsy-12*	G	A	**Yes**
	9927293	V	*C05H2.13*	T	A	**Yes**
	7548600	V	*ftn-1*	G	A	**Yes**
	8101405	V	*str-90*	G	A	**Yes**
	7377580	V	*C03G6.1*	A	G	**Yes**
	9059201	V	*C25E10.13*	C	T	**Yes**
	7403427	V	*srj-13*	A	G	**Yes**
	8657771	V	*ZC190.4*	C	T	**Yes**
	6889638	V	*unc-70*	C	G	**Yes**
	6956711	V	*mec-1*	T	C	**Yes**
	8646873	V	*F28A12.4*	C	T	**Yes**
	9707449	V	*xbx-6*	T	C	**Yes**
	7860248	V	*C37C3.1*	A	T	**Yes**
	9663159	V	*B0331.2*	T	G	**Yes**
	6889637	V	*unc-70*	G	N/A^a^	**Yes**
	10397711	V	*ugt-1*	C	C	No
	7245105	V	*C05C8.7*	C	G	**Yes**
	7524635	V	*C54F6.6*	C	T	**Yes**
	8122360	V	*D1014.6*	A	A	Yes
	8758179	V	*B0507.7*	G	G or A^b^	**N/A**
	6956743	V	*mec-1*	C	N/A^c^	**Yes**
	6302464	V	*sago-1*	A	N/A^d^	**Yes**
	8684563	V	*cln-3.3*	A	A	Yes
	8122376	V	*D1014.6*	A	A	Yes
	7430567	V	*srw-71*	C	T	**Yes**
	9376379	V	*E02C12.10*	G	T	**Yes**
	9662868	V	*B0331.2*	T	C	**Yes**
	10234235	V	*str-182*	C	T	**Yes**
	9340210	V	*K07B1.8*	C	C	Yes
	10067798	V	*F57A8.4*	A	C	**No**
	6956744	V	*mec-1*	G	N/A^b^	**Yes**
	9928614	V	*T07C12.12*	C	T	**Yes**
	7704134	V	*srw-3*	C	N/A^e^	**No**
	7367493	V	*C03G6.20*	C	C	Yes
	9026669	V	*R03H4.7*	G	G	Yes
	8482100	V	*T23B12.10*	C	C	Yes
	9301469	V	*F44A2.5*	G	G	Yes
	6712439	V	*sna-1*	T	N/A^e^	**No**
	8454246	V	*T23B12.11*	C	C	Yes
	8358352	V	*F38E1.9*	T	T	Yes
	9720648	V	*F40F5.9*	C	C	Yes
	7226632	V	*gei-6*	A	A	Yes
	10158311	V	*srsx-30*	G	G	Yes
	8277551	V	*str-136*	T	T	Yes

N/A, not applicable. Bold words in last column reflect positions at which a sequence change was predicted by ‘galign’.

a alteration predicted by Deletion_search; sequenced change is a G-to-C substitution.

b galign read both wild-type and mutant sequences here. The mutant sequence was confirmed by sequencing.

c alteration predicted by Deletion_search; sequenced change is a C-to-G substitution.

d alteration predicted by Deletion_search; sequenced change is a A-to-G substitution.

e alteration predicted by Deletion_searc.

Sarin *et al*. previously determined the sequences of 54 positions on chromosome V containing potential single-nucleotide substitution polymorphisms in the *lsy-12*(*ot177*) strain [1], showing that 33 of these positions had true polymorphisms. ‘galign’ predicted 32/33 confirmed polymorphisms in this strain, including the causal lesion leading to the *lsy-12*(*ot177*) phenotype (Table 1). 28/33 were detected using SNP_search, and 4/33 were found by Deletion_search. One position confirmed by Sarin *et al*. as mutant was read as wild type by SNP_search. Of the 20 positions confirmed by Sarin *et al*. not to contain base alterations, ‘galign’ assigned 19/20 as wild-type, with one position flagged as a potential mutation site by Deletion_search. In the same paper, Sarin *et al*. also documented 26 insertions/deletions in the *ot177* strain. As shown in Table 2, ‘galign’ identified 25/26 of these lesions. 16/25 were identified as regions with no reads, 6/25 were identified as nucleotide substitution calls, and 3/25 had both.

**Table 2:** Prediction of deletions and insertions by ‘galign’.

Posiiton on Chromosome V	Known lesion^a^	‘galign’ WT reads^b^	‘galign’ Mutant reads	Detected by ‘galign’?	Comments^c^
9113930	Insertion of G	1	2	Yes	
10336187	Deletion of T	0	0	Yes	8 no-read nts
10304793	Insertion of C	15	1	Yes	
8233594	Insertion of G	0	0	Yes	6 no-read nts
9698443	Insertion of C	0	0	Yes	14 no-read nts
6865466	Insertion of G	0	0	Yes	16 no-read nts
9290037	Insertion of G	1	1	Yes	
8425798	Insertion of C	0	0	Yes	6 no-read nts
8757751	Insertion of A	0	1	Yes	Adjacent 7 no-read nts
9911365	Insertion of A	3	2	Yes	
10245639	Insertion of A	0	1	Yes	Adjacent 6 no-read nts
7205626	Deletion of G	0	0	Yes	16 no-read nts
9669523	Insertion of C	19	1	Yes	
6873249	Insertion of C	0	0	Yes	16 no-read nts
7725858	Deletion of GATC	0	0	Yes	2 no-read nts
8276295	Insertion of A	0	0	Yes	11 no-read nts
9007888	Deletion of T	2	0	No	
6972719	Insertion of T	0	3	Yes	Adjacent 7 no-read nts
8969821	Insertion of T	0	0	Yes	7 no-read nts
10067797	Insertion of C	1	3	Yes	
7386791	Insertion of T	0	0	Yes	13 no-read nts
10235807	Deletion of T	0	0	Yes	17 no-read nts
9155855	Insertion of T	0	0	Yes	3 no-read nts
9750975	Deletion of G	0	0	Yes	8 no-read nts
10164059	Insertion of C	0	0	Yes	9 no-read nts
9309038	Deletion of T	0	0	Yes	15 no-read nts

a Lesions were described by Sarin *et al*. [1].

b Reads are at the positions adjacent to deletion or insertion site.

c no-read nts, nucletides with no sequence reads surrounding the deletion/insertion site.

Together with the *daf-6* studies, these results suggest that ‘galign’ is able to detect sequence alterations reliably.

### *In silico* Validation of ‘galign’

**Figure 6:**
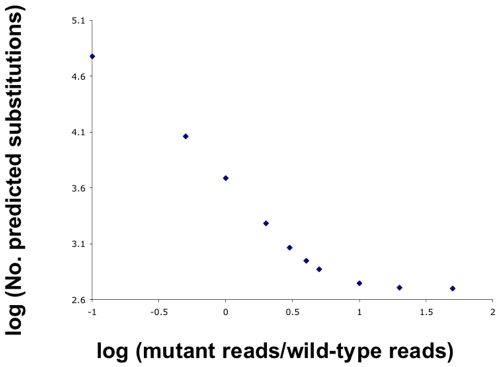
Empirical correlation of the number of SNP_search-predicted nucleotide substitutions with SNP_search parameters. The number of predicted nucleotide substitutions generated by SNP_search using the *lsy-12*(*ot177*) data [1] is shown as a function of the minimal ratio of the number of mutant and wild-type reads sufficient to trigger a SNP call. This ratio is a user-specified parameter. Numbers on each axis are the base 10 log of the actual values.

To extend our validation studies of ‘galign’ we examined the *lsy-12*(*ot177*) sequence reads data set, and compared exonic single nucleotide substitution predictions between ‘galign’ and the program ‘Maq’ (version 0.7.1). Both ‘galign’ and ‘Maq’ outputs are highly dependent on the specific filtering parameters used. For example, ‘galign’ will predict 1916 genomic polymorphisms for the *lsy-12*(*ot177*) data set for mutant/wild-type reads ratio of 2, and 1163 polymorphisms for a ratio of 3 (see Figure 6). For our analysis we used the default settings of each program, which for ‘galign’ required a mutant/wild-type reads ratio of 3, and a minimum mutant read of 2. For ‘Maq’, we used the “SNPfilter” function with its default settings requiring a minimum read depth of 3 reads to identify what the software considered reliable reads. In addition, ‘Maq’ was allowed to align reads with up to 2 mismatches. The alignments were performed on a Mac OSX Version 10.4.11 2×2.66 Ghz Dual-Core Intel Xeon machine, with 6 Gb 667 Mhz DDR2 FB-DIMM RAM. ‘galign’ performed the alignment and analysis in 103 minutes, consistent with our analysis of the *daf-6* data set, which required 75 minutes. Alignment and analysis on ‘Maq’ was completed in 238 minutes and did not include data on lesion positions with respect to exons, did not display neighboring sequence context, and did not predict amino-acid changes. Secondary processing of ‘Maq’ data was done using custom perl scripts to analyze the data (see Materials and Methods).

‘galign’ identified 1163 putative nucleotide substitutions, whereas ‘Maq’ identified 516. Of these, 256 predictions were shared between both programs, 907 were unique to ‘galign’ and 260 were unique to ‘Maq’. Both ‘galign’ and ‘Maq’ alignment tools correctly identified the lesion leading to the *lsy-12* phenotype. The substitutions that were unique to ‘Maq’ fell into the following categories. 181/260 predictions corresponded to positions that ‘galign’ read as having more wild-type than mutant reads, making it unlikely that most of these correspond to *bona fide* changes. To test this we sequenced 7 of these ‘Maq’-predicted polymorphisms and found that 7/7 were false positives. 19/260 predictions unique to ‘Maq’ were found as having no wild-type or mutant reads by the Deletion_search tool of ‘galign’. 29/260 predictions had a ratio of mutant to wild-type reads of less than 3, but greater than 1. One of these was sequenced and found to be a false positive. Finally, 31/260 predictions corresponded to ones identified by the ‘galign’ alignment tool to contain one mutant and zero wild-type reads, and could correspond to true lesions. Thus, at least 80% of the polymorphisms uniquely identified by ‘Maq’ using its default settings are likely to be false positives.

As with ‘Maq’, 482/907 of the polymorphisms uniquely identified by ‘galign’ had 2 or more wild-type reads, and were thus also likely to be false positives; however, the remaining reads seemed likely to encode genuine polymorphisms. We sequenced 8 of these and showed that 3/8 indeed had a nucleotide substitution, suggesting that like ‘Maq’, roughly 80% of all predictions unique to galign may be false positives. Although the percentage of genuine mutations identified by ‘Maq’ and ‘galign’ is similar, the absolute number of true polymorphisms predicted uniquely be ‘galign’ may be over 3.5× more than predicted by ‘Maq’ under the assay conditions.

Together with the direct sequencing results, these *in silico* studies support the notion that ‘galign’ is a reliable tool for sequence polymorphism discovery.

## Discussion

The ‘galign’ software described here presents a number of improvements compared to available Illumina/Solexa polymorphism-detection tools. First, the output files of ‘galign’ allow immediate access to the locations and sequence alterations revealed in a sequencing experiment. This allows researchers to sift through large amounts of data in a straight-forward manner. Second, the application is significantly faster than the commonly used program ‘Maq’. In our case, ‘galign’ performed the alignment and processed the data nearly 2.5 times faster than ‘Maq’. In practice, this should allow a mutagenized *C. elegans* genome to be queried for polymorphisms leading to a phenotype in less than two hours. For larger genomes, the difference in time becomes, of course, more pronounced. Part of the speed difference is likely attributable to the alignment algorithm itself. However, some of the difference may also be accounted for by the additional information used by ‘Maq’ in its analysis. Specifically, in predicting sequence alterations, ‘Maq’ takes into account the quality scores provided for each nucleotide in a sequence read- a feature not incorporated into ‘galign’, and which in principle should increase prediction accuracy, as quality scores reflect the degree of confidence assigned to every nucleotide call. Our comparison of ‘galign’ and ‘Maq’ on the same data set suggests, however, that quality scores do not significantly add to polymorphism identification. Indeed, ‘galign’ was able to predict nearly all sequence-confirmed polymorphisms detected by ‘Maq’. Instead, it seems that an important determinant of accurate polymorphism calling is the ratio of mutant to wild-type reads at a given position. It is possible that quality scores are of greater value when the number of sequence reads is low; however, in such instances stochastic effects become important, and it is unclear whether more accurate reads can properly counter random sequencing errors. For identifying phenotypically relevant mutations, a reasonable approach aimed at cost saving might be to use only one or two sequencing lanes, and test whether the data are sufficient to generate candidate lesions. If not, additional lanes can be sequenced.

Although ‘galign’ predictions are generally accurate, additional improvements are likely to increase the performance of the analysis software. Illumina/Solexa sequencing can now be performed in a paired-end mode, in which the sequence of each deposited fragment can be read from both ends. Such information has predictive value in the case of deletions or insertions, and is generally more reliable with increasing lesion size. ‘galign’ currently is not designed to take these data pairs into account, except as independent sequence reads. Incorporation of paired-end data should be particularly useful in estimating the sizes of insertions. In addition, it is likely that as the technology develops, accurate Illumina sequence reads beyond 78 nucleotides will become commonplace, and so modification of the ‘galign’ Format_convert utility to accommodate these developments will be important.

The memory requirements of ‘galign’ may pose difficulties in applying the program to genome sizes larger than those of *C. elegans* or *Drosophila*. For the human genome, it is estimated that around 50 Gb of memory are needed for analysis using the current settings of ‘galign’. Given that memory usage affects processing speed, a computer with significantly larger memory capacity would be required to run the program at maximal speeds. Although such requirements are not prohibitive, they may pose a challenge for incidental users of the software. However, these requirements are unlikely to be significantly alleviated by any polymorphism-detection tool, as storage of information for every nucleotide in the genome is a minimal algorithmic requirement. It is unlikely that fewer than 24 Gb of memory could be used for a genome the size of the human genome, unless speed is compromised, byte architecture is manipulated, or data compression is employed.

Finally, ‘galign’ is currently implemented to detect sequence reads containing only zero or one mismatch. Manipulation of the algorithm to include additional mismatches would, in principle, allow utilization of more sequence reads, reducing the number of sequence fragments needed for accurate polymorphism prediction. Indeed, the average number of reads per nucleotide for ‘galign’ on the *ot177* data set was 9, whereas for ‘Maq’, which allows more mismatches, was 14. It is worth noting, however, that ‘Maq’ predictions seem to carry a higher false positive rate than ‘galign’ predictions, a difference that may be due to inaccurate alignments of multiply mismatched sequence reads. If this is the case, the stringency of ‘galign’ may contribute to fewer false positives of this class.

## Materials and Methods

### Software Access

‘galign’ software, source code, and user information can be downloaded at no cost from http://shahamlab.rockefeller.edu/galign and is subject to the GNU General Public License. Information on program installation is provided at http://shahamlab.rockefeller.edu/galign/galign.htm, as is a step-by-step protocol for performing a polymorphism analysis of the *C. elegans* genome.

### Converting ‘Maq’ Data to ‘galign’ Format

To compare polymorphisms predicted by ‘Maq’ to those predicted by ‘galign’ we used a perl script to scan the ‘Maq’ SNPfilter file to obtain all positions called as polymorphic. These positions were then converted to the ‘galign’ alignment tool output format with each position receiving a mutant/wild-type ratio of 15, greater than the detection threshold for the default settings of ‘galign’ and guaranteeing that all sequences were read. This new file was then fed into SNP_search for assignment to exons.

### Accessory files

Information to generate accessory files for ‘galign’ was downloaded from www.wormbase.org using the WormMart module. Exon, intron, and intergenic region position data were sorted in order of chromosomes, and from left to right using a perl script. Exon frame information is not directly available from WormBase, and was calculated using a perl script comparing each exon coding start site to its position in the cDNA. Source codes for these scripts are available at “shahamlab.rockefeller.edu/galign/accessory_files_source_codes”.

### Sequencing

Sequences of polymorphic regions were confirmed by generating 300 bp PCR fragments surrounding the lesion examined, and subjecting these to Sanger sequencing.
